# Epidemiology of multi-drug resistant Tuberculosis in the western region of Kenya

**DOI:** 10.3934/microbiol.2024014

**Published:** 2024-04-22

**Authors:** George O Lugonzo, Ezekiel M. Njeru, William Songock, Albert A. Okumu, Eric M. Ndombi

**Affiliations:** 1 Department of Biochemistry, Microbiology, and Biotechnology, Kenyatta University, Nairobi, Kenya; 2 Department of Medical Microbiology and Parasitology, Kenyatta University, Nairobi, Kenya; 3 Centre for Global Health Research, Kenya Medical Research Institute, Kisumu, Kenya

**Keywords:** tuberculosis, HIV, isoniazid, rifampicin, multidrug-resistant tuberculosis, risk factors, western Kenya

## Abstract

Multidrug-resistant tuberculosis (TB) (MDR-TB), or TB that is simultaneously resistant to both isoniazid (INH) and rifampicin (RIF), is a barrier to successful TB control and treatment. Stratified data on MDR-TB, particularly in the high-burden western Kenya region, remain unknown. This data is important to monitor the efficacy of TB control and treatment efforts. Herein, we determined the molecular epidemiology of drug-resistant TB and associated risk factors in western Kenya. This was a non-experimental, population-based, cross-sectional study conducted between January and August 2018. Morning sputum samples of individuals suspected of pulmonary TB were collected, processed, and screened for *Mycobacterium tuberculosis* (Mtb) and drug resistance using line probe assay (LPA) and *Mycobacterium* growth indicator tubes (MGIT) culture. MGIT-positive samples were cultured on brain heart infusion (BHII) agar media, and the presence of Mtb was validated using Immunochromatographic assay (ICA). Drug sensitivity was performed on MGIT and ICA-positive but BHI-negative samples. Statistical significance was set at *P* < 0.05. Of the 622 Mtb isolates, 536 (86.2%) were susceptible to RIF and INH. The rest, 86 (13.83%), were resistant to either drugs or both. A two-sample proportional equality test revealed that the MDR-TB prevalence in western Kenya (5%) did not vary significantly from the global MDR-TB estimate (3.9%) (P = 0.196). Men comprised the majority of susceptible and resistant TB (75.9% and 77.4%%, respectively). Also, compared with healthy individuals, the prevalence of HIV was significantly higher in MDR-TB patients (35.9% vs 5.6%). Finally, TB prevalence was highest in individuals aged 25–44 years, who accounted for 58.4% of the total TB cases. Evidently, the prevalence of MDRTB in western Kenya is high. Particular attention should be paid to men, young adults, and those with HIV, who bear the greatest burden of resistant TB. Overall, there is a need to refine TB control and treatment programs in the region to yield better outcomes.

## 1. Introduction

Despite numerous local and global control measures, such as Directly Observed Therapies (DOTs), TB remains a global public health concern. For instance, in 2019, over 10 million TB cases were reported, resulting in over 1.2 million deaths. Intriguingly, an estimated 25% of the world's TB deaths occur in the African Region. Poverty and high HIV/AIDs burden are among the contributing factors for high TB cases in Africa [1]. Kenya ranks 15^th^ among 22 high-burden TB countries globally, where HIV prevalence is estimated at between 5.6% and 6.3%, but with significant regional variation [2],[3]. Particularly, TB notification in Kenya increased from 50 cases per 100,000 individuals in 1990 to 558 (455–662) cases per 100,000 adult population [4]. In western Kenya, the prevalence of TB ranges from 127 per 100,000 individuals in Bungoma County to 426 per 100,000 individuals in Homa Bay County.

Multidrug-resistant tuberculosis (MDR-TB) infections are simultaneously resistant to both Isoniazid (INH) and Rifampicin (RIF), which has confounded the fight against TB [5]. Its treatment is very complicated, expensive (almost six times more expensive than susceptible TB), and toxic to the body, and because the therapy lasts between 18–24 months, it complicates treatment compliance, which mostly leads to poor treatment outcomes [6]. Thus, globally, only 54% of patients with MDR-TB undergo successful treatment [3]. Primary resistance occurs when a treatment-naive individual contracts an already resistant bacillus strain, whereas acquired resistance develops during TB chemotherapy [7]. Control of TB infection and drug resistance are constrained by late diagnosis, inefficient TB case management, poor drug choice and adherence, suboptimal treatment, weak TB management programs, migration, poverty, and overcrowding, among other factors [8],[9]. Although the global incidence of TB has been declining by 1.4% per year between 2000−2014 and by 1.9% between 2015 and 2016 [10], that of MDR-TB has been rising [11]. For instance, the number of individuals with multidrug-resistant TB increased by more than 20% between 2009 and 2016, mainly attributed to poor drug choice and adherence [8],[11]. Even though a 2018 WHO report [3] revealed that the incidences of MDR-TB in new and previously treated patients were 3.5% and 18%, respectively, down from 4.1% and 19% in 2016 [12], the prevalence remains high. According to the drug resistance survey of 2015 by Kenya's Ministry of Health, the prevalence of MDR TB in the country among previously treated and new cases is 2.1% and 0.7%, respectively [13].

In Kenya, the MDR-TB prevalence is highly variable, ranging from 0.4% in Mombasa [14] to 0.8 in Nairobi [15] and as high as 6% in western Kenya among smear-positive individuals [16]. Even so, reports on the recent MDR-TB prevalence for TB patients in the western region of Kenya, regardless of smear results, remain scanty. TB diagnosis in Kenya at the hospital level is mainly performed using microscopy and imaging. Other methods, including culture tests and MTB Rif Assay (GeneXpert) are used in advanced hospitals and reference laboratories. Even though sputum smear microscopy is cheap, it is less sensitive when the bacterial load is below 10,000 organisms/mL of sputum sample [17], while, relative to culture, chest radiographs fail to detect 9% of positive TB cases [18]. On the other hand, the culture test is more sensitive, but it is slow and expensive [19], meaning that it could be unaffordable to some patients or delay treatment initiation. Overall, about 40% of TB cases in Kenya go unnoticed and thus untreated, which accelerates the spread of the disease [20]. The global aim of WHO is to reduce 95% of TB-related deaths and 90% of TB incidences relative to 2015 rates and ensure 0% catastrophic costs due to TB by 2035 [21]. To achieve these, an accurate and up to date burden of TB needs to be determined. Routine resistance surveillance is critical for creating and refining approaches to controlling the emergence of new strains, spreading resistance genes, and guiding clinical decisions regarding appropriate treatments. Even so, little work has been done in the high-burden western region of Kenya to understand the evolving epidemiology of the tuberculosis epidemic and the associated factors that can be addressed to mitigate the burden of TB. Previously, six counties (Kisumu, Migori, Homabay, Siaya, Kisii and Nyamira) of western Kenya had been found to collectively contribute to about 20% of TB cases notified in the country [22]. In the present study, we investigated the epidemiology of MDR-TB in the western region of Kenya. Additionally, given that young age, HIV infection, and male sex have been found to be risk factors for TB infections [23], we also investigated the relationship between these factors and MDR-TB prevalence in the western region of Kenya. This study was inspired by the paucity of up-to-date data on RIF and INH resistance, the key first line tuberculosis drugs. The findings of this study are important in assessing the progress toward the ambitious “end TB strategy” by WHO, which aims at reducing the incidence of tuberculosis (TB) by 90% and deaths due to TB by 95% by 2035 relative to the 2015 figures, as well as eliminating the economic and social burden associated with this disease [24].

## 2. Materials and methods

### 2.1. Study site and sampling design

**Figure 1.**
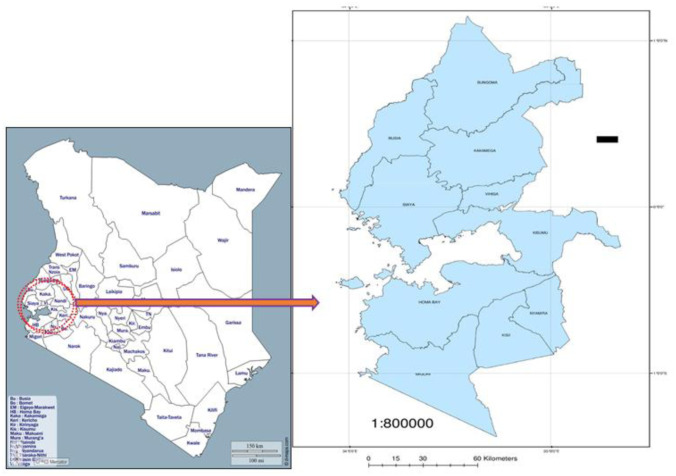
Map of Kenya with the western region shown in the circle. (https://d-maps.com/infos.php?lang=en)

Currently, Kenya is divided into 47 counties. The present study involved 10 counties on the western side of Kenya: Busia, Bungoma, Kakamega, Vihiga, Siaya, Kisumu, Homa Bay, Migori, Kisii and Nyamira. According to the 2019 Kenya National Bureau of Statistics (KNBS) census, the 10 counties had a combined population of 11,291,422 people, up from 9,776,993 people in 2009 [25]. This was a non-experimental, population-based, cross-sectional study. Between January and August 2018, patients presenting with pulmonary tuberculosis (PTB) symptoms in 100 selected health facilities (10 per county) in western Kenya were enrolled in the study based on randomized cluster sampling (Figure 1). These were general health facilities with the capacity to diagnose and treat TB. Diagnosis in these centres is mainly through smear microscopy and radiography. Patients with any confirmed underlying respiratory disease(s), younger than five years, and recent residents in the region (less than 5-year continuous stay) were all excluded from the study. The protocol for this research was approved by the Kenyatta University Ethical Review Committee (KUERC) (PKU/2040/11187) and the Kenya Medical Research Institute's Scientific and Ethics Review Unit (KEMRI-SERU). All participants above the age of 18 years consented to participate in this study in writing. Consent for those below 18 was obtained from their guardians.

### 2.2. Analysis of samples

#### 2.2.1. Sputum collection and HIV testing

At least 1 mL of early morning sputum samples from patients presenting with suspected TB symptoms were collected and transported to the KEMRI-CDC CHGR tuberculosis laboratory between 2–8 °C and processed within 24 hours of collection. Voluntary HIV testing was performed at the respective health facility using the uni-gold HIV rapid test (Trinity Biotech PLS. Brey, Co. Wicklow, Ireland) following the manufacturer's instructions. Positive results were further validated using the Abbot Determine ® HIV-1/2 test (Abbot Laboratories, Chicago, Illinois, USA) according to the manufacturer's instructions. Data such as name, age, gender of the patient, contact details, type of suspected TB, history of TB or contact with TB individuals, and status of risk for TB were also captured at baseline. Data for all patients was handled with confidentiality and was only shared with authorized persons.

#### 2.2.2. Sample processing

Sputum samples were processed using the N-acetyl-l-cysteine-Sodium hydroxide (NALC-NaOH) decontamination procedure [26]. Briefly, sputum samples were decontaminated and processed using an equal volume of 0.25 g of NALC in 50 mL of 4% sodium hydroxide (NaOH) solution and 2.9% sodium citrate (Na_3_C_6_H_5_O_7_). Phosphate buffer (PBS) was added after 20 minutes of digestion and decontamination to stop the reaction. The samples were then centrifuged at 3000 g for 15 minutes, where the pellets were re-suspended in 2 mL of PBS.

#### 2.2.3. Zielh Neelsen and fluorescent microscopy and MGIT culture

Zielh Neelsen (ZN) staining (BD, Ziehl-Neelsen staining kit) and fluorescent microscopy (FM) (BD brand fluorescent staining kit) were performed using respective kits for preliminary identification of Mtb bacilli following the manufacturer's instruction. Moreover, the same samples were cultured in liquid *Mycobacterium* growth indicator 960 tubes (Becton Dickinson, Oxford Science Park, Oxford, UK) following the manufacturer's instructions. Briefly, 0.5 mL (500 µL) of the samples were inoculated in MGIT tubes supplemented with polymixin, Amphotericin B, nalidixic acid, trimethoprim, and Azloxacillim (PANTA) in 800 µL of *Mycobacteria* growth supplement (SIRE supplement). The MGIT tubes contain 7.0 mL of modified Middlebrook 7H9 broth base and were incubated at 37 °C in MGIT BACTECK 960 instrument for a maximum of 42 days. Since Mtb is a slow-growing *Bacillus*, any growth detected in less than 24 hours was presumed to be contamination. Such samples were re-decontaminated with 4% sodium hydroxide (NaOH) and re-incubated as described in the preceding section. Negative and positive controls were performed alongside each test/batch using artificial sputum and *Mycobacterium tuberculosis* strain H37Rv, respectively.

#### 2.2.4. Validation of Mtb in positive MGITs

ZN staining was performed on MGIT samples flagged positive by the instrument. The tubes were further observed visually. The Mtb particles appear granular at the bottom of the tubes, but turbidity indicates contamination. Culturing was performed for a maximum of 42 days. In addition, MGIT and ZN-positive samples were streaked on brain heart infusion agar (BHI) media and incubated for up to 48 hours at 37 °C. Because Mtb does not grow on BHI, growth within this period was treated as contamination. Meanwhile, an Immunochromatographic test (ICT), a rapid technique for Mtb identification, was performed on MGIT and ZN-positive but BHI-negative samples as previously described [27].

#### 2.2.5. Drug susceptibility testing (DST)

Drug susceptibility testing of MGIT, ZN, and ICT-positive but BHI-negative isolates within 1–5 days after detection of growth in MGITs was performed as previously described [28] using the BACTEC MGIT 960 system. The method relies on silicone, a fluorescent compound, at the bottom of the MGIT tubes. The compound is sensitive to oxygen. Initially, there is a large amount of dissolved oxygen, which decreases as the *Mycobacterium* grows, increasing the fluorescence. The change in the fluorescence intensity is measured as the growth value (GV). When a certain level of fluorescence is reached, the instrument interprets a tube as positive. Briefly, Mtb isolates were serially diluted with normal saline at the ratio of 1:5 before inoculation of 100 µL of Mtb to MGITs containing 800 µL of growth supplement mixed with 0.1 µg/mL of INH or 1.0 µg/mL of RIF. DST was performed against INH and RIF alone, being the only drugs of interest. For the negative control, 5 mL phosphate buffer was used, whereas Mtb strain H37Rv diluted in the ratio of 1:500 was used as the positive growth control. Incubation was done for a maximum of 13 days. The instrument interprets results when the growth unit (GU) in the positive control tube reaches 400. Growth of more than 100 units was indicative of resistance.

### 2.3. Line probe assay

Line Probe Assay (LPA) was performed to identify Mtb loci and the mutations associated with the RIF and INH resistance. Mtb DNA was extracted from the decontaminated samples using the GenoLyse® kit (Hain Lifescience GmbH, Nehren, Germany), following the manufacturer's instructions. The extracted DNA was amplified through 20 cycles in the PCR system 9700 (Hain Lifescience, Bandol, France). Hybridization was finally performed to screen for specific Mtb genes and *rpoB, KatG*, and *inhA* gene mutations using the GenoType®MTBDRplus kit (Hain Lifescience GmbH, Nehren, Germany). The technique involves an initial stage of DNA strand separation before probing with complementary strands.

### 2.4. Data analysis

Data were analyzed using SPSS software V. 20 (IBM SPSS Statistics for Windows, Version 20.0. Armonk, NY: IBM Corp). Significant differences between groups were analyzed using chi-square tests, whereas the relationships between developing MDR-TB resistance and selected demographic characteristics were assessed using Pearson's correlation with Cramer's V value used to quantify the relationships. *P* ≤ 0.05 was considered statistically significant.

## 3. Results

### 3.1. Demographics

In general, 3654 individuals were enrolled in this study. Of these, 622 participants were positive for pulmonary TB, of which 472 (75.9%) were males and 150 (24.1%) were females. The individuals ranged from 6 to 81 years old, with a mean age and standard deviation of 37.71 and 13.217 years, respectively. Of the 622 individuals, 192 (30.9%) were HIV positive, whereas 338 (54.3%) were HIV negative. Meanwhile, the HIV status of 92 (14.8%) individuals was unknown because they declined to be tested. Among the HIV-positive individuals, 145 (75.5%) were males, whereas 47 (24.5%) were females. These statistics are summarized in Table 1.

**Table 1.** Demographic characteristics of the tuberculosis patients evaluated in this study.

Characteristics	Number of patients (%)
Age (years)	
≤14	11 (1.8)
15–24	81 (13.0)
25–34	194 (31.2)
35–44	169 (27.2)
45–54	89 (14.3)
55–64	51 (8.2)
65–74	24 (3.9)
75–84	3 (0.5)
Range	6–81 (75) years
Mean age	37.71 years
Gender	
Male	472 (75.9%)
Female	150 (24.1%)
HIV status	
Negative	338 (54.3)
Positive	192 (30.9)
Unknown	92 (14.8)
HIV gender distribution	
Male	145 (75.5)
Female	47 (24.5)

HIV-Human immunodeficiency virus.

### 3.2. Multidrug resistance

**Figure 2.**
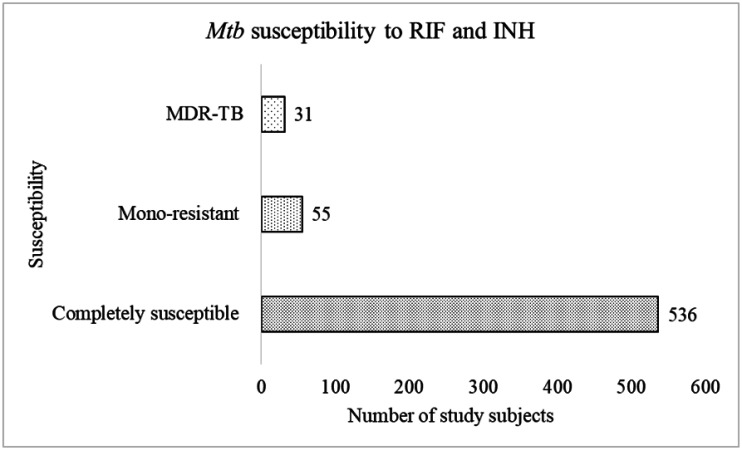
Graph of *Mycobacterium tuberculosis* susceptibility and resistance of the 622 individuals with tuberculosis.

Of the 622 Mtb isolates, 536 (86.2%) were susceptible to both RIF and INH. The rest, 86 (13.83%), were resistant to at least RIF or INH or both, while which 26 (4.2%) and 29 (4.7%) were RIF and INH monoresistant, respectively. Meanwhile, 31 (4.9%) clinical isolates were resistant to both RIF and INH (MDR). These statistics are summarized in Figure 2. The majority of MDR-TB individuals were aged below 34 years. Regarding MDRTB along gender, 24 (77.4%) of the 31 MDR-TB cases were males, whereas only 7 (22.6%) were females. For HIV status, 35.5% of the individuals with MDR-TB were HIV positive, 58.1% were HIV negative, and 6.5% declined to get tested for HIV. The two-sample test for equality of proportions revealed that the MDR-TB prevalence was not significantly higher than the global MDR-TB estimate (3.9%) (*P = 0.196*; CI: 95%).

At the county level, the highest TB cases were observed in Homabay county (21.5%), Siaya (19.3%), and Migori county (15.1%), but were lowest in Buia county (2.4%), Vihiga county (2.6%), and Kisii county. For MDR-TB, the highest cases were observed in Homabay (22.6%), Siaya (16.1%), and Kisumu (12.9%), and were least in Busia (3.2%), Vihiga (3.2%), and Bungoma (6.5%). The highest rate of developing MDR-TB within a county was observed in Kisii county (13.0%), Busia county (6.7%), and Kakamega county (6.5%). These findings are summarized in Table 2.

**Table 2.** The geographical distribution of the TB cases among the counties in western Kenya.

	County										Total

	Nyamira	Bungoma	Busia	Homabay	Kakamega	Kisumu	Migori	Kisii	Vihiga	Siaya	
Susceptible	53	45	14	127	29	82	91	20	15	115	591
MDRTB	3 (5.4%)	2 (4.3%)	1 (6.7%)	7 (5.2%)	2 (6.5%)	4 (4.7%)	3 (3.2%)	3 (13.0%)	1 (6.2%)	5 (4.2%)	31 (100%)
Total	56	47	15	134	31	86	94	23	16	120	622

Susceptible: Patients with susceptible or monoresistant TB; Patients with multi-drug resistant tuberculosis.

### 3.3. Risk factors for developing MDR-TB resistance

Univariate analysis revealed that, overall, sex, age, and HIV infection do not increase the risk of developing MDR-TB (95% CI). These findings are summarized in Table 3.

**Table 3.** The relationship between sex, age, and HIV status and developing MDR-TB (n = 622).

		Resistance		OR	95% C.I		P

		Mono/Sus. TB (%)	MDR-TB (%)		Lower	Upper
Sex	Male	448 (94.9)	24 (5.1)	Ref			
	Female	143 (95.3)	7 (4.7)	0.9	0.4	2.2	0.8
HIV Status	Negative	320 (94.7)	18 (5.3)	Ref			
	Positive	181 (94.3)	11 (5.7)	1.1	0.5	2.3	0.197
	Declined	90 (97.8)	2 (2.2)	0.4	0.1	1.7	0.219
Age (Years)	≤14	6 (66.7)	3 (33.3)	7.4	1.4	38.7	0.018
	15–24	74 (93.7)	5 (6.3)	Ref			
	25–34	179 (94.7)	10 (5.3)	0.8	0.3	2.5	0.736
	35–44	172 (96.6)	6 (3.4)	0.5	0.2	1.7	0.287
	45–54	87 (95.6)	4 (4.4)	0.7	0.2	2.6	0.576
	55–64	47 (96.0)	2 (4.1)	0.6	0.1	3.4	0.590
	65+	26 (96.3)	1 (3.7)	0.6	0.1	5.1	0.615

(Mono/sus. TB: mono resistant or susceptible TB, MDR-TB multi-drug resistant tuberculosis).

## 4. Discussion

This research investigated the epidemiology of drug-resistant TB in western Kenya and its likely risk factors. In general, 622 Mtb isolates were recovered from an equal number of patients. Of these, 536 (86.2%) were completely susceptible to both RIF and INH. Of the remaining 86 (13.83%), 26 (4.2%) and 29 (4.7%) were RIF and INH monoresistant, respectively, and 31 (4.9%) were multidrug-resistant (MDR). Across the counties, the MDR-TB rate ranged from 3.2% in Migori county to 13.0% in Kisii, although the majority were between 4%–6%. In many regions, INH resistance has been found to be higher than RIF resistance [29]. Even though isoniazid preventive therapy (IPT) has been proposed for this phenomenon [30], a separate meta-analysis revealed conflicting findings [31]. As such, why INH monoresistance is higher than RIF mono-resistance is not clear [29]. The MDR-TB prevalence in western Kenya was comparable to the global estimates of 3.9% and other countries in Africa with similar TB disease structures [32]–[36]. However, it differed from a similar study in the same region by Ogwang et al. that largely focused on drug resistance in HIV-positive and negative individuals and resistance to a variety of TB drugs [37]. For the present study, all individuals presenting with TB symptoms were sampled, resulting in a large sample size, irrespective of the smear-positive results. Additionally, the sampling in the present study was completely random but was multi-stage in the Ogwang et al. study.

In the present study, most TB patients were males (75.9%), with females only accounting for 24.1% of the cases, consistent with existing findings reported in the last two decades [12],[38]. Similar trends have been reported in India [39] and Ethiopia [40]. The higher prevalence of TB in men has been mainly attributed to behavioral factors such as smoking, alcohol consumption [41], and delay in seeking treatment [42]. Herein, within sex, 5.1% and 4.7% of men and women were diagnosed with MDR-TB, respectively. The risk of infection following TB exposure is primarily governed by exogenous factors and is determined by an intrinsic combination of the infectiousness of the source case, proximity to contact, and social and behavioral risk factors, including smoking, alcohol, and indoor air pollution. Although more men comprised the majority of MDR-TB, this research did not find any association between sex and developing MDR-TB as previously reported [43]. Regarding counties, the highest TB and corresponding MDR-TB cases were observed in Homabay County.

According to the National AIDS Control Council (NACC) report, as of 2016, the overall prevalence of HIV in Kenya was 5.9% and over 15% in some regions in western Kenya [44]. Herein, the prevalence of HIV among individuals with MDR-TB was 35.5%, while the prevalence of MDR-TB in TB patients living with HIV was 5.7%. Although Ramaswamy and Musser (1998) [45] and van Halsema et al. (2012) [46] found an association between HIV infection and the development of drug resistance, this study reports dissimilar findings (CI: 95%; P = 0.05). Our findings are, however, consistent with separate reports in Benin [47], Burkina Faso [48], and Mali [49], with the prevalence of HIV in MDR-TB individuals being more than 11 times that of the general population in the latter country. In Kenya, TB is treated using the antituberculosis regimens recommended by the World Health Organization [6]. Regarding the treatment regimen for MDR-TB, the latest WHO guidelines prioritize a new 6-month regimen consisting of bedaquiline (B), pretomanid (Pa), linezolid (L), and moxifloxacin (M), referred to as BPaLM [50].

In high TB-burden settings, HIV infection is the most important risk factor to develop active TB [51]. Particularly, HIV infection accelerates the reactivation of latent TB or increases the susceptibility of developing Mtb or re-infection. HIV/AIDS increases the risk of developing active TB mainly by killing CD4^+^ T cells in the blood, lymphoid tissues, and mucosa [52]. However, separate evidence showed that susceptibility to TB increases soon after HIV infection, even before significant CD4^+^ T-cells decrease below 500 cells/µL [53], demonstrating the complex relationship between HIV infection and the development of active TB. Even though several cross-sectional studies have not found an association between HIV infection and MDR-TB, two meta-analyses have shown that HIV infections indeed increase the risk of developing primary MDR-TB [54],[55], and this may result from the small sample sizes used in the meta-analyses. Overall, compared to the general population, the prevalence of HIV in MDR-TB individuals is very high.

In this study, the prevalence of MDR-TB was highest in the age group between 25–34 years (32.3) and those between 35–44 years (19.4%). The prevalence of HIV in TB patients in the two age groups was 31.3% and 34.3%, respectively. However, overall, age was not a risk factor for developing MDR-TB, comparable to previous findings [56],[57]. Young people commonly develop infectious forms of tuberculosis and frequently have a much wider range of social contacts outside the household [58]. Consequently, as well as suffering from the disease, adolescents and young adults with tuberculosis contribute to ongoing transmission. High incidences of drug resistant (DR-TB) in young people and young adults have been reported in India (18–25 years), Nigeria (15–29 years), and Ethiopia [59]–[61]. This higher rate of DR-TB in the active age groups might be attributed to frequent movement, increased outdoor contact, and higher case notification due to greater health awareness among this group of people. In general, despite the comparable MDRTB prevalence in western Kenya and global estimates, the burden of drug-resistant TB remains high, particularly in the context of WHO 2030 TB targets to significantly reduce 95% of TB-related deaths and 90% of TB incidences relative to 2015 rates and ensure 0% catastrophic costs due to TB by 2035. Particular attention should be paid to men, young adults, and those with HIV who bear the greatest burden of resistant TB in western Kenya.
